# Angiotensin-converting enzyme 2/angiotensin-(1–7)/Mas axis activates Akt signaling to ameliorate hepatic steatosis

**DOI:** 10.1038/srep21592

**Published:** 2016-02-17

**Authors:** Xi Cao, Fangyuan Yang, Tingting Shi, Mingxia Yuan, Zhong Xin, Rongrong Xie, Sen Li, Hongbing Li, Jin-Kui Yang

**Affiliations:** 1 Department of Endocrinology, Beijing Tongren Hospital, Capital Medical University, Beijing 10730, China; 2 Beijing Key Laboratory of Diabetes Research and Care, Beijing 100730, China

## Abstract

The classical axis of renin-angiotensin system (RAS), angiotensin (Ang)-converting enzyme (ACE)/Ang II/AT1, contributes to the development of non-alcoholic fatty liver disease (NAFLD). However, the role of bypass axis of RAS (Angiotensin-converting enzyme 2 (ACE2)/Ang-(1–7)/Mas) in hepatic steatosis is still unclear. Here we showed that deletion of ACE2 aggravates liver steatosis, which is correlated with the increased expression of hepatic lipogenic genes and the decreased expression of fatty acid oxidation-related genes in the liver of *ACE2* knockout (*ACE2*^−/y^) mice. Meanwhile, oxidative stress and inflammation were also aggravated in *ACE2*^−/y^ mice. On the contrary, overexpression of ACE2 improved fatty liver in db/db mice, and the mRNA levels of fatty acid oxidation-related genes were up-regulated. *In vitro*, Ang-(1–7)/ACE2 ameliorated hepatic steatosis, oxidative stress and inflammation in free fatty acid (FFA)-induced HepG2 cells, and what’s more, Akt inhibitors reduced ACE2-mediated lipid metabolism. Furthermore, ACE2-mediated Akt activation could be attenuated by blockade of ATP/P2 receptor/Calmodulin (CaM) pathway. These results indicated that Ang-(1–7)/ACE2/Mas axis may reduce liver lipid accumulation partly by regulating lipid-metabolizing genes through ATP/P2 receptor/CaM signaling pathway. Our findings support the potential role of ACE2/Ang-(1–7)/Mas axis in prevention and treatment of hepatic lipid metabolism.

Non-alcoholic fatty liver disease (NAFLD) is one of the most common chronic liver disorders worldwide^1^. As a possible model for the pathogenesis of NAFLD, the “two hit hypothesis” is widely accepted. The “first hit” is accumulation of lipid in the liver, which makes the liver more sensitive to “second hit”, such as oxidative stress, endotoxins, or abnormal cytokine productions, which can cause inflammation and/or eventual fibrosis of the liver^2^,^3^.

The classical axis of renin-angiotensin system (RAS), angiotensin (Ang)-converting enzyme (ACE)/Ang II/AT1, has been shown to contribute to the development of NAFLD^4^,^5^. Ang II causes development and progression of NAFLD in the transgenic Ren2 rat model (with elevated tissue Ang II) by increasing hepatic reactive oxygen species (ROS)^5^. Increased activation of both systemic and local RAS has been noted in the patients with cirrhosis^6^ and chronic hepatitis C^7^. The blockage of the RAS significantly ameliorates hepatic fibrogenesis in animal models induced by methionine choline deficient diet, CCl4 or bile duct ligation^8^,^9^,^10^.

Recently, we and Santos *et al.* reported that the ACE2/Ang-(1–7)/Mas axis counteracted against ACE/Ang II/AT1axis in the liver^11^,^12^. We demonstrated for the first time that ACE2 knockout (*ACE2*^−/y^) mice exhibit progressive impairments in glucose tolerance and reduced first-phase insulin secretion and that ACE2 maybe a new target for the treatment of type 2 diabetes^13^,^14^. More recently, we reported that activation of the ACE2/Ang-(1–7)/Mas axis led to improved hepatic insulin resistance through the Akt/PI3K/IRS-1/JNK insulin signaling pathway^12^. On the other hand, loss of ACE2 activity worsens liver fibrosis in chronic liver injury models, and administration of recombinant ACE2 shows therapeutic potential^15^. Although the functions of ACE2/Ang-(1–7) in liver insulin resistance and liver fibrosis are established, the role it plays in NAFLD is still unknown.

ACE2 is expressed in the liver^16^, which is one of the major organs regulating triglyceride (TG) and cholesterol metabolism. However, to date, there is no published study using a genetic model with excessive activity of ACE2/Ang-(1–7)/Mas axis to directly evaluate its role on hepatic steatosis. In this study, we provide physiological and molecular evidences for ACE2/Ang-(1–7)/Mas axis improvement in hepatic steatosis and provide new insights and understanding into possible hepatic lipid metabolism mechanisms of ACE2/Ang-(1–7)/Mas axis.

## Results

### Deletion of ACE2 Aggravates the Development of Hepatic Steatosis in *ACE2*^*−/y*^ Mice

**Figure 1:**
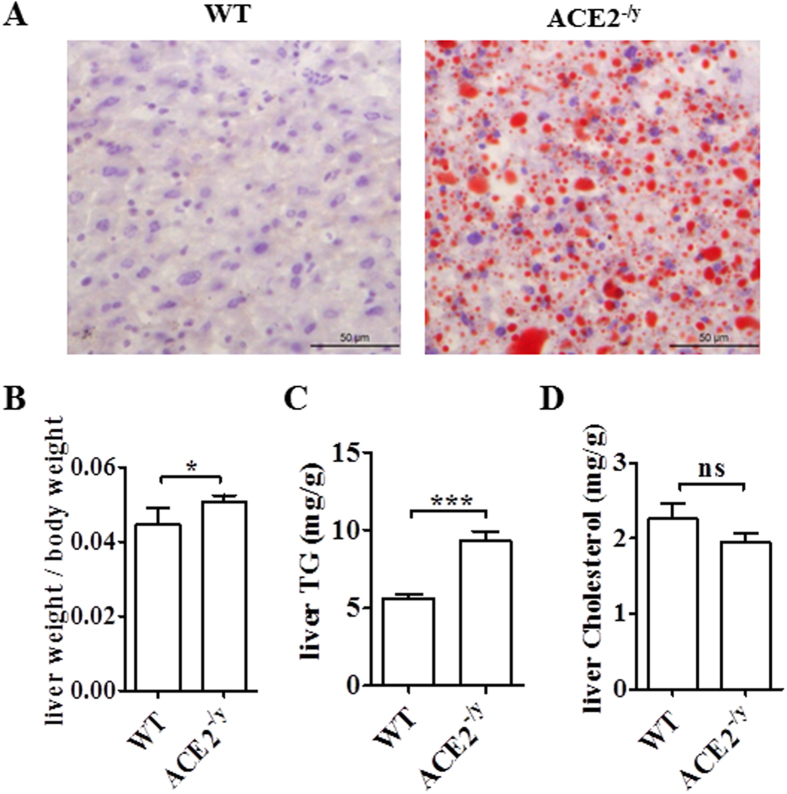
The absence of ACE2 aggravated the development of hepatic steatosis in *ACE2*^−/y^ mice. (**A**) Representative images of liver sections from WT and *ACE2*^−/y^ mice stained with Oil-Red-O (n = 5/each group). (**B–D**) The liver weight, hepatic triglyceride and cholesterol levels were determined (n = 8/each group). *P < 0.05 and ***P < 0.001 versus WT by Student’s t test.

*ACE2*^−/y^ mice developed a prominent fatty liver phenotype featuring a pale liver appearance caused by extensive fat accumulation. Results from the histological examination and Oil-Red-O staining of hepatic sections showed an extensive hepatic lipid deposition in the livers of *ACE2*^−/y^ mice and a reduced accumulation in wild-type (WT) mice livers (Fig. 1A). Consistently, liver weights and liver TG contents were significantly higher in *ACE2*^−/y^ mice than in WT mice (Fig. 1B,C), but the cholesterol contents did not change significantly (Fig. 1D).

### Deletion of ACE2 Aggravates Hepatic Steatosis, Oxidative Stress and Inflammation in *ACE2*^*−/y*^ Mice

**Figure 2:**
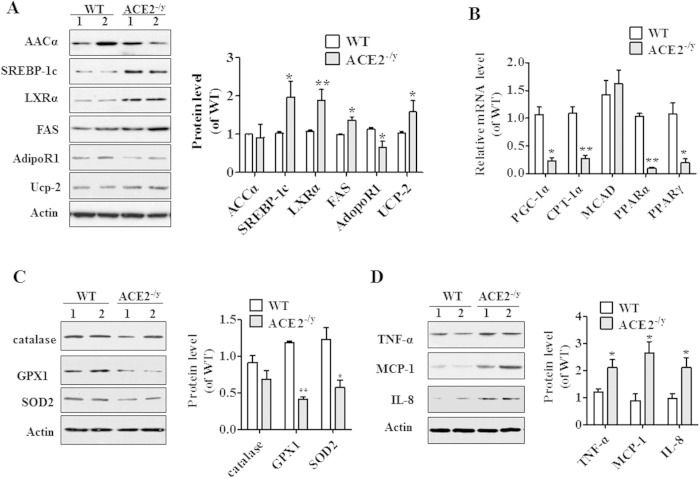
ACE2 regulated hepatic gene expressions involved in lipid-metabolizing, oxidative stress and inflammation in the liver of *ACE2*^−/y^ mice. (**A**) Relative protein levels of lipid-metabolizing (ACCα, SREBP-1c, LXRα, FAS, AdipoR1 and UCP-2). (**B**) Relative gene expression levels of fatty acid oxidation-related genes (*PGC-1α*, *CPT-1α*, *PPARα*, *PPARγ* and *MCAD*). (**C**) Relative protein levels of oxidative stress signaling (catalase, GPX1 and SOD2), and (**D**) inflammation (TNF-α, MCP-1 and IL-8). The data are presented as the mean ± SD of n = 4 independent experiments in *ACE2*^−/y^ mice. *P < 0.05 and **P < 0.01 versus WT mice by Student’s t test.

The protein levels of lipid-metabolizing genes, including sterol regulatory element-binding protein-1c (SREBP-1c), acetyl-CoA carboxylase α (ACCα), liver X receptor-α (LXRα), fatty acid synthase (FAS) and uncoupling protein-2 (UCP-2) were up-regulated, whereas AdipoR1 was repressed in *ACE2*^−/y^ mice (Fig. 2A). Meanwhile, the mRNA levels of fatty acid oxidation-related genes, *carnitine palmitoyltransferase Iα* (*CPT-1α*), *peroxisome proliferator-activated receptor alpha* (*PPARα*), *PPAR gamma* (*PPARγ*) and *PPARγ coactivator 1α* (*PGC-1α*) were down-regulated, and little change was observed in *medium chain acy -CoA dehydrogenas* (*MCAD*) in the liver of *ACE2*^−/y^ mice (Fig. 2B). These results suggested that deletion of ACE2 may aggravate hepatic steatosis in *ACE2*^−/y^ mice.

The absence of *ACE2* also regulates the expressions of genes related to oxidative stress and inflammation. Marker genes for oxidative stress signaling, as glutathione peroxidase (Gpx1), catalase and superoxide dismutase 2 (SOD2) were reduced in *ACE2*^−/y^ mice (Fig. 2C). The expressions of proinflammatory cytokines, as tumor necrosis factor (TNF)-α, monocyte chemotactic protein-1 (MCP-1) and interleukin-8 (IL-8) were increased in *ACE2*^−/y^ mice (Fig. 2D). These results indicated that deletion of ACE2 may increase oxidative stress and inflammation in the liver of *ACE2*^−/y^ mice.

### Overexpression of ACE2 Ameliorates Hepatic Steatosis in db/db Mice

**Figure 3:**
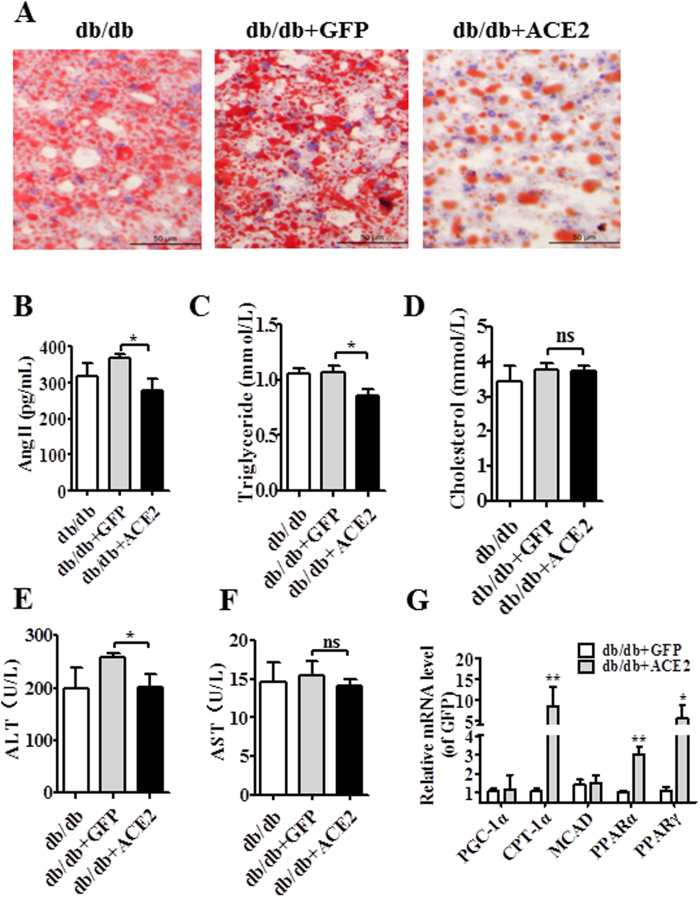
Hepatic overexpression of ACE2 ameliorated hepatic steatosis of db/db mice. (**A**) Representative images of liver sections from db/db, db/db + GFP and db/db + ACE2 mice stained with Oil-Red-O. Quantitative assay of (**B**) AngII, (**C**) triglyceride and cholesterol, (**D**) ALT and AST levels in ACE2-overexpressing db/db mice. *P < 0.05versus db/db + GFP mice (n = 6). (**E**) Relative gene expression levels of fatty acid oxidation-related genes (*PGC-1α*, *CPT-1α*, *PPARα*, *PPARγ* and *MCAD*). *P < 0.05 versus db/db + GFP by Student’s t test.

To further investigate the effect of ACE2 *in vivo*, ACE2 was overexpressed in the liver of db/db mice to evaluate its role in lipid metabolism. Compared with Ad-GFP-treated mice, Ad-ACE2-treated mice exhibited a significant reduction in fat accumulation in the liver (Fig. 3A). Moreover, serum Ang II, TG and Alanine aminotransferase (ALT) levels were decreased by hepatic ACE2 overexpression (Fig. 3B–D), with little change in serum cholesterol and Aspartate aminotransferase (AST) levels (Fig. 3C,D). The amelioration of plasma lipid profiles after ACE2 overexpression suggested that ACE2 might affect whole-body insulin sensitivity. To test whether ACE2 improved glucose metabolism, a glucose tolerance tests (GTT) was performed. Compared with Ad-GFP-treated mice, insulin sensitivity was significantly improved in Ad-ACE2-treated mice (Supporting Fig. S1A, S1B).

Next, we investigate the expression levels of lipid metabolism-related genes. The mRNA levels of fatty acid oxidation-related genes, *CPT-1α*, *PPARα* and *PPARγ* were up-regulated, while little change was observed in *PGC-1α* and *MCAD* mRNA levels in the liver of Ad-ACE2-treated mice (Fig. 3E). We also wondered whether ACE2 alters the expression of lipogenesis genes. However, the expression of lipogenesis genes did not exhibit significant difference between Ad-GFP-treated and Ad-ACE2-treated groups (Supporting Fig. S1C). All together, these results indicated that ACE2 could significantly improve hepatic steatosis in db/db mice.

### Treatment of Ang-(1–7)/ACE2 Ameliorates FFA-induced Hepatic Steatosis in HepG2 Cells through Akt

**Figure 4:**
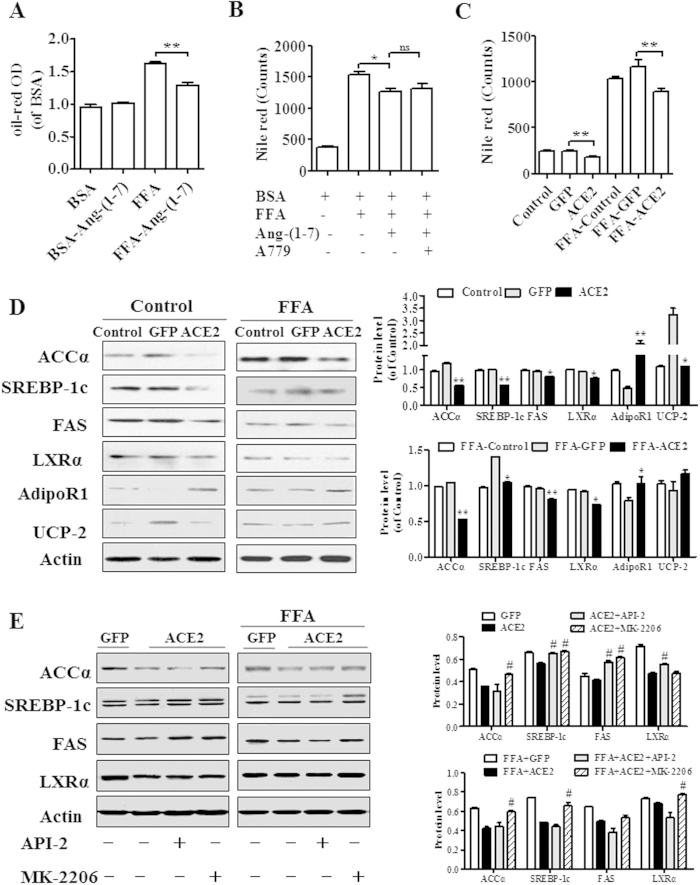
Ang-(1–7)/ACE2 ameliorated hepatic steatosis in FFA-induced HepG2 cells. Cells were exposed to 2 mM of FFA (2:1, oleate: palmitate) for 24 h. FFA-induced hepatic steatosis in HepG2 cell treated with Ang-(1–7) or A779. (**A**) Analyze lipid accumulation by Oil Red O staining. (**B**) Intracellular lipids were stained with Nile red, and the fluorescence intensity of Nile red was measured by flow cytometry. (**C**) Intracellular lipids content analysis following ACE2 overexpression. (**D**) Relative protein levels of ACCα, SREBP-1c, LXRα, FAS, UCP-2 and AdipoR1 in ACE2-overexpressing HepG2 cells and FFA-induced HepG2 cells. (**E**) Relative protein levels of ACCα, SREBP-1c, LXRα and FAS in API-2 or MK-2206-treated ACE2-overexpressing HepG2 cells. Cells were treated with API-2 (20 μM) and MK-2206 (10 μM) for 24 h. The data are presented as the mean ± SD of n = 3 independent experiments in HepG2 cells. *P < 0.05 and **P < 0.01 versus GFP group by one-way ANOVA; ^#^P < 0.05 versus ACE2 group by one-way ANOVA.

To investigate the influence of Ang-(1–7) on hepatic steatosis, we treated HepG2 cells with FFA to induce an *in vitro* cellular steatosis model. As revealed by Oil-Red-O staining, FFA induced substantial lipid accumulation in HepG2 cells, while Ang-(1–7)-treated HepG2 cells significantly prevented lipid accumulation (Fig. 4A). The similar result was also detected by Nile-red in Ang-(1–7)-treated cells, while Mas receptor antagonist (A779) did not counteract the effect of Ang-(1–7) on lipid accumulation (Fig. 4B).

To further explore these findings, we then investigated the effect of ACE2 in FFA-induced HepG2 cells. The expression of ACE2 in ACE2-overexpressing HepG2 cells was detected (Supporting Fig. S2). Similarly, lipid accumulation was significantly suppressed in control or FFA-induced ACE2-overexpressing HepG2 cells (Fig. 4C).

To test whether Ang-(1–7)/ACE2 has any effect on fatty acid metabolism-related proteins, we examined ACCα, SREBP-1c, LXRα, FAS, UCP-2 and AdipoR1 in ACE2-overexpressing HepG2 cells. ACCα, SREBP-1c, LXRα, FAS and UCP-2 were up-regulated, whereas AdipoR1 was decreased (Fig. 4D). Consistently, the expression of ACCα, SREBP-1, LXRα and FAS were inhibited, while AdipoR1 was increased in FFA-induced ACE2-overexpressing HepG2 cells (Fig. 4D). These results indicated that Ang-(1–7)/ACE2 could ameliorate hepatic lipid accumulation.

To detect whether the inhibitory effects of ACE2 on lipid accumulation are regulated by Akt, ACE2-overexpressing HepG2 cells were treated with Akt inhibitors (Triciribine (API-2) and MK-2206). Interestingly, the expression of ACCα, SREBP-1c, LXRα and FAS were increased in API-2-treated and MK-2206-treated groups (Fig. 4E). These results indicated that treatment of ACE2-overexpressing HepG2 cells with Akt inhibitors may reduce ACE2-mediated lipid metabolism *in vitro*.

### Treatment of Ang-(1–7)/ACE2 Inhibits FFA-Induced Oxidative Stress and Inflammation in HepG2 Cells

**Figure 5:**
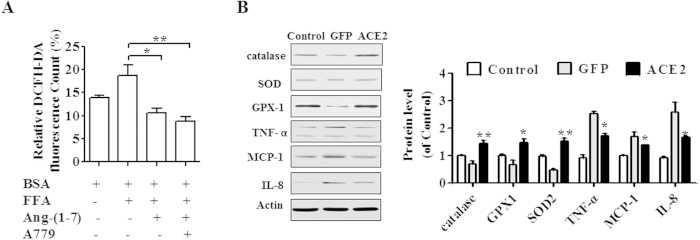
Ang-(1–7)/ACE2 inhibit oxidative stress and inflammation in FFA-induced HepG2 cells. (**A**) The levels of intracellular ROS were stained with DCF-DA and the fluorescence intensity of DCF-DA was measured by flow cytometry. (**B**) Relative protein levels of anti-oxidative stress related proteins (catalase, GPX1 and SOD2) and inflammation related proteins (TNF-α, MCP-1 and IL-8) in ACE2-overexpressing HepG2 cells. The data are presented as the mean ± SD of n = 3 independent experiments in HepG2 cells. *P < 0.05 and **P < 0.01 versus control or empty vector by one-way ANOVA.

We also measured the levels of intracellular ROS using DCF-DA in FFA induced HepG2 cells. We observed a significant decrease in intracellular ROS in Ang-(1–7)-treated FFA-induced HepG2 cells. Mas receptor antagonist A779 partly counteracted the effect of Ang-(1–7) on ROS production in HepG2 cells (Fig. 5A). These results indicated that Ang-(1–7) decreases ROS production in FFA-induced HepG2 cells.

The western blot results further documented that ACE2 regulated the expressions of genes related to oxidative stress signaling and inflammation. The expressions of marker genes for oxidative stress signaling (Gpx1, catalase and SOD2) were increased, while proinflammatory cytokines (TNF-α, MCP-1 and IL-8) were reduced (Fig. 5B). These results suggested that ACE2 could protect against oxidative stress and inflammation in HepG2 cells.

### Overexpression of ACE2 Activates Akt through the ATP/P2 receptor/CaM Pathway

**Figure 6:**
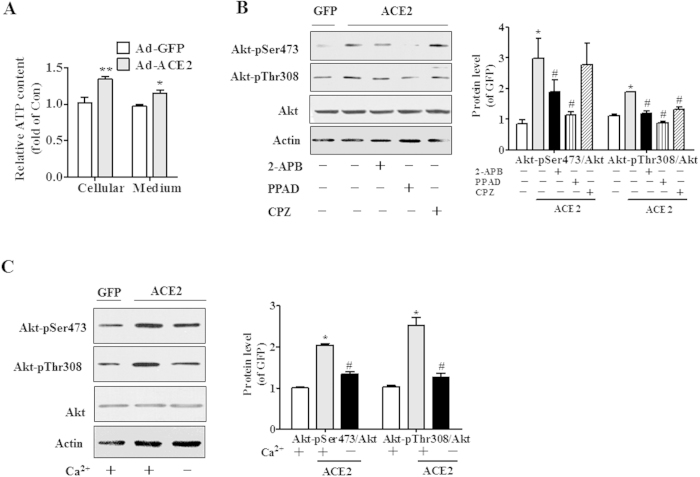
ACE2 activated Akt through the ATP/P2 receptor/CaM signal pathway. (**A**) The intracellular and extracellular ATP levels in ACE2-overexpressing HepG2 cells. (**B**) ACE2-induced Akt activation was inhibited by the IP3R antagonist (2-APB), the ATP receptor P2 antagonist (PPADS), and the CaM antagonist (CPZ). Infected HepG2 cells were treated with 2-APB (10 μM), PPADS (50 μM) or CPZ (100 μM) for 1 hour before pAkt levels was measured. The data are presented as the mean ± SD of n = 3 independent experiments in HepG2 cells. *P < 0.05 versus control cells, ^#^P < 0.05 versus Ad-ACE2 infected cells without 2-APB, PPAD or CPZ treatment (n = 3). (**C**) ACE2-induced Akt activation was partially dependent on the presence of extracellular calcium. Infected cells were treated with calcium free medium HANK’S for 2 hours before pAkt levels were assayed. The data are presented as the mean ± SD of n = 3 independent experiments in HepG2 cells. *P < 0.05 versus control cells; #P < 0.05 versus Ad-ACE2 infected cells with calcium (n = 3).

Considering Ang II can induce production of mitochondrial reactive oxygen species and *ACE2*^−/y^ mice displayed elevated levels of oxidative stress^12^,^17^, the impact of ACE2 on mitochondria function was examined by detecting cellular ATP content. Expectedly, ACE2 overexpression elevated intracellular and extracellular ATP levels in HepG2 cells (Fig. 6A).

To further explore these findings, we then investigated the ATP/P2 receptor/CaM signaling pathway in ACE2-overexpressing HepG2 cells. ACE2-induced Akt activation was repressed by the inositol 1,4,5-trisphosphate receptor (IP3R) antagonist (2-APB) and the calmodulin (CaM) antagonist (CPZ) in HepG2 cells (Fig. 6B). Importantly, ACE2-induced Akt phosphorylation was completely abolished by the ATP receptor P2 antagonist (PPADS) in HepG2 cells (Fig. 6B). In addition, ACE2-induced Akt activation was also partially dependent on the presence of extracellular Ca^2+^ (Fig. 6C). These results suggested that ACE2-induced Akt activation is partly dependent on ATP/P2 receptor/CaM signaling pathway.

## Discussion

The classical pathway of RAS, ACE/AngII/AT1, modulates and contributes to the development of NAFLD^4^,^5^,^8^. On the other hand, Ang-(1–7) exerts an important role of anti-obesity by Mas receptor^11^,^18^,^19^,^20^. The present study is the first to clarify the possible mechanisms of ACE2/Ang-(1–7)/Mas regulate hepatic steatosis. We show that deletion of ACE2 aggravated hepatic steatosis, oxidative stress and inflammation in *ACE2*^−/y^ mice. On the contrary, overexpression of ACE2 improved hyperglycemia and fatty liver in db/db mice. *In vitro* study, Ang-(1–7)/ACE2 ameliorated hepatic steatosis, oxidative stress and inflammation in FFA-induced HepG2 cells. Notably, Akt inhibitors reduced ACE2-mediated lipid metabolism, and what’s more, ACE2-mediated Akt activation can be attenuated by blockade of ATP/P2 receptor/CaM pathway. Taken together, Ang-(1–7)/ACE2/Mas axis may reduce liver lipid accumulation partly by regulating lipid-metabolizing genes through ATP/P2 receptor/CaM signaling pathway. The reduction of oxidative stress and inflammation may also be involved in the amelioration of hepatic steatosis.

Multiple metabolic pathways lead to the development of hepatic steatosis, including increased lipogenesis and lipolysis, and decreased fatty acid oxidation^21^. Key transcriptional regulators such as LXRα and SREBP-1c coordinately control lipogenesis^22^. LXRα and SREBP-1c increase the expression of key lipogenic genes, including those for FAS, SCD1 and ACC^23^. ACC1 converts acetyl-CoA to malonyl-CoA, and inhibits fatty acid entry into the mitochondria reducing β-oxidation. FAS utilizes both acetyl-CoA and malonyl-CoA to form palmitic acid (C16:0). Besides, adiponectin appears to have a pivotal role in improving fatty acid oxidation and decreasing fatty acid synthesis^24^. The liver has adiponectin receptors, and their stimulations lead to increased fatty acid β-oxidation and thereby decreased hepatic TG content. Aberrant induction of these factors may contribute to hepatic steatosis. In the present study, the changes of these genes in *ACE2*^−/y^ mice and ACE2-overexpressing HepG2 cells support the idea that ACE2 ameliorates hepatic steatosis.

Several studies demonstrated that the excessive production of reactive oxygen species and altered redox balance promoted hepatic lipid accumulation^5^,^25^,^26^. Our previous study documented that the ROS levels were increased in the liver of *ACE2*^−/y^ mice, whereas in HepG2 cells, Ang-(1–7) could protect against oxidative stress by inhibiting NADPH oxidase expression^12^. Acting to protect against oxidative stress is a complex system of enzymatic antioxidants (SOD, GPX, glutathione reductase, catalase) and non-enzymatic antioxidants (glutathione (GSH), vitamins C and D)^27^. In the present study, the increased lipid accumulation in the liver may be involved in the decrease in the expressions of enzymatic antioxidants genes and the resulting increased ROS production. The expression of oxidative stress signaling in *ACE2*^−/y^ mice and FFA-induced ACE2-overexpressing HepG2 cells were in agreement with previous data.

Numerous studies have reported an association between NAFLD and systemic inflammatory stress^28^,^29^. Steatosis is a necessary step in the pathogenesis of NAFLD, but inflammation is required to bring about steatohepatitis, which leads to chronic damage to hepatocytes and progressive fibrosis. It has been demonstrated that elevated proinflammatory cytokines expression, as TNF-α, promoted hepatic lipid accumulation^30^. Several studies showed that many RAS components are expressed in the liver meddling metabolic and inflammatory processes^5^,^31^,^32^,^33^. The increased expression of Ang II induces NAFLD and modulates inflammatory cell recruitment into the liver during liver injury^4^,^5^. In addition, recent study has shown RAS as a potent mediator in the activation of inflammatory mechanisms involved in obesity^18^,^31^. Our current findings are consistent with these data and document for the first time that the inflammatory levels increased in the liver of *ACE2*^−/y^ mice, whereas in HepG2 cells, ACE2 could protect against inflammatory stress by inhibiting proinflammatory cytokines expression.

It has been previously reported that reduced hepatic ATP levels are associated with the development of NAFLD in rats^34^. ATP has been demonstrated to be a signaling molecule to activate the PI3K/Akt signaling pathway^35^,^36^,^37^. The ATP receptors (P2X and P2Y receptors) mediate such action. P2X receptors are permeable to calcium, which are ligand-gated ion channels^37^. P2Y receptors are G-protein-coupled receptors which stimulate PLC to increase IP3 production, resulting in calcium release from internal stores^37^. Increased cytosolic free calcium activates CaM, leading to the activation of the PI3K/Akt pathway^38^,^39^. In the present study, we found that the P2 receptor antagonists (PPADS) completely abolished ACE2 induced Akt activation, which reveals a critical role of ATP receptors in this process. Furthermore, Akt activation was also significantly blocked by IP3R antagonist (2-APB), CaM antagonist (CPZ) and depletion of extracellular calcium in HepG2 cells. These data reveal the possible roles of ATP/P2 receptor/CaM signal pathway in ACE2–mediated hepatic insulin resistance and liver lipid accumulation in HepG2 cells. What is more, these findings offer another plausible explanation for the molecular basis of the relationship between the ACE2/Ang-(1–7)/Mas axis and liver glucose/lipid metabolism.

Insulin resistance and steatosis is closely associated in the development of NAFLD^40^. Elevated hepatic FFAs worsen insulin resistance. The accompanying increase in oxidative stress and proinflammatory cytokine production following liver lipid accumulation causes a further increase in insulin resistance, thereby establishing a vicious cycle^3^. Fatty acid synthesis cross-talks with insulin signaling, and Akt plays a pivotal role in glucose and lipid metabolism signaling pathway^41^. Akt has been found to not only regulate gluconeogenesis and glycogenolysis, but also play a critical role in the regulation of liver lipid metabolism. Activation of Akt represses lipogenesis and prevents excessive lipid deposition in the liver^42^,^43^,^44^. In addition, the increased phosphorylation level of Akt in the liver is associated with the amelioration of steatosis in diabetic mice^45^. In our more recently research on the effect of ACE2/Ang-(1–7)/Mas axis on hepatic insulin resistance, the phosphorylation levels of Akt increased markedly in ACE2-overexpressing cells, while significantly inhibited in the liver of *ACE2*^−/y^ mice. As expected, Akt inhibitors can reduce ACE2-mediated lipid metabolism. Furthermore, hepatic overexpression of ACE2 improved hyperglycemia and fatty liver in db/db Mice. Together with these two studies, the mechanism underlying glucose and lipid metabolism could involve the ability of ACE2/Ang-(1–7) to activate Akt.

One of the limitations of the current study is that we did not get positive results from high fat-induced *ACE2*^−/y^ mice (data not shown), these may due to the new finding function of ACE2 as a key regulator of dietary amino acid homeostasis, innate immunity, gut microbial ecology, and transmissible susceptibility to colitis^46^, which may affect the metabolism of high fat diet. These need to be confirmed in further systematic experiment; maybe hepatic specific knockout ACE2 mice can solve this issue.

**Figure 7:**
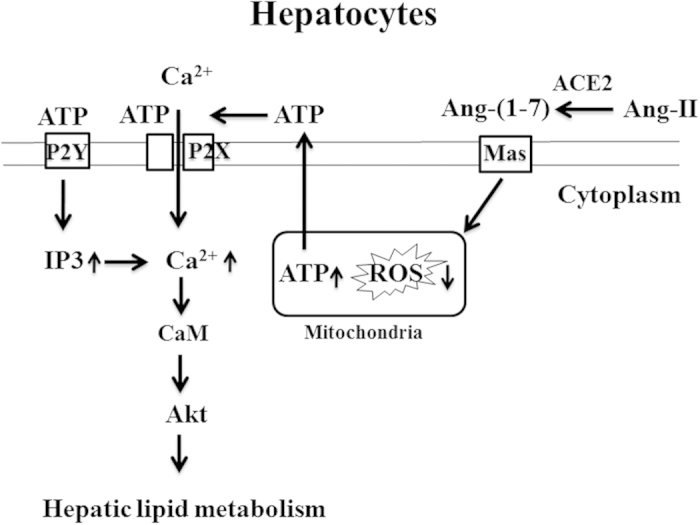
Mechanisms involved in ACE2/Ang-(1–7)/Mas axis activation-induced improvement of hepatic lipid metabolism. The up- or down-regulation of metabolic pathways is indicated by arrows (↑ for up-regulation and ↓ for down-regulation).

In summary, as described in Fig. 7, our study demonstrates that ACE2/Ang-(1–7)/Mas axis activation plays an important role in hepatic lipid metabolism through a mechanism that could involve regulation of lipid-metabolizing genes through ATP/P2 receptor/CaM signaling pathway, and reduction of oxidative stress and inflammation. Together with our more recently research on ACE2/Ang-(1–7)/Mas axis can inhibit hepatic insulin resistance, our current findings suggest that ACE2/Ang-(1–7)/Mas axis is a potential target for drug therapy for NAFLD.

## Methods

### Animal

*ACE2* knockout (ACE2 KO, *ACE2*^−/y^) mice were a gift from Prof. Dr. Josef Penninger from Institute for Molecular Biotechnology GmbH. In every experiment, ACE2 KO mice were identified by PCR on genomic DNA extracted from tail biopsies. Male db/db mice at the age of 6 weeks were purchased from Nanjing biological medicine research institute affiliated to Nanjing medical university. db/db mice were maintained on the BKS background. ACE2 KO mice were identified by PCR using genomic DNA extracted from tail biopsies. All of the mice were maintained on a 12 h light/dark cycle. All of the experiments in this study were performed using male *ACE2*^−/y^ mice and their age- and sex-matched wild-type (WT) littermates, and were fed with standard chow.

All animals were handled in accordance with the protocol approved by the Ethics Committee of Animal Research at Beijing Tongren Hospital, Capital Medical University, Beijing, China.

### Cell Culture and Drugs

HepG2 cells (Cell Resource Center, IBMS, CAMS/PUMC) were cultured in DMEM (Hyclone) containing 10% fetal bovine serum (FBS) (Hyclone), 100 U/mL penicillin and 0.1 mg/mL streptomycin. Cells were maintained at 37 °C with humidified air at 5% CO_2_ and passaged every 2 days by trypsinization. *In vitro* model of cellular steatosis, palmitic acid (C16:0) and oleic acid (C18:1) (Sigma, St. Louis, MO) were dissolved in isopropanol to obtain 20 or 40 mM stock mixture solution (2:1 oleate: palmitate), and the concentration of vehicle was 1% in final incubations. Cells were exposed to 1 or 2 mM of free fatty acids (FFA) for 24 h.

Akt inhibitor of Triciribine (API-2) and MK-2206 were purchased from Selleck Chemicals, and dissolved with DMSO. Cells were treated with API-2 (20 μM) and MK-2206 (10 μM) for 24 h.

### Overexpression of ACE2 in HepG2 cells and Mouse Liver

To overexpress *ACE2* in the liver, the adenovirus coding for rat *ACE2* (rACE2) upstream of an enhanced green fluorescent protein (eGFP) reporter gene (Ad-rACE2-eGFP) or with the eGFP virus alone (Ad-eGFP) were purchased from SinoGenoMax (Beijing, China), and were injected into db/db mice (male, 5 to 7-week-old) by way of the tail vein (5 × 10^8^ particle forming units (pfu) in a total volume of 100 μL of 0.9% wt/vol saline). At the 6th day post-virus injection, glucose tolerance tests (GTT) were performed. On the 7th day, the fed animals were sacrificed for experimental analysis. To over express ACE2 in HepG2 cells, HepG2 cells were infected with 1.0 × 10^4^ pfu Ad-ACE2 or Ad-GFP for 48 hours.

### Biochemical Assays

Serum triglyceride, cholesterol, aspartate aminotransferase and alanine aminotransferase levels were measured using enzymatic kits (Biosino, China) by automatic biochemical analyzer (Hitachi 7160, Japanese). Ang II in serum was measured by radioimmunoassay (BEIJING SINO-UK INSTITUTE OF BIOLOGICAL TECHNOLOGY, china).

### RNA Extraction and Quantitative Real-time RT-PCR

Total RNA was isolated using TRIzol reagent (Invitrogen, Carlsbad, CA, USA) according to the manufacturer’s instructions. A total of 500ng of RNA was used as the template for the first-strand cDNA synthesis using ReverTraAceqPCR RT Kit (TOYOBO, Osaka, Japan) in accordance with the manufacturer’s protocol. The transcripts were quantified using Light Cycler 480Real-Time PCR system (Roche, Basel, Switzerland). Primers were designed using Primer Quest (Integrated DNA Technologies, Inc).

### Histological Analysis

The tissue samples were fixed in 10% formaldehyde for 1 hour at 4 °C, and the fixed specimens were then dehydrated and embedded in paraffin according to standard procedure. The specimens were cut into 5μm thick, and stained with Oil-Red-O for lipid accumulation. The sections were then observed with a microscope (200 × ) and analyzed on four random fields/slides.

### Determination of ROS Levels

The dye 2′,7′-dichlorofluorescin diacetate (DCF-DA) (Sigma-Aldrich, St Louis, Mo., USA) was used to detect intracellular ROS production by cells. The fluorescence of this cell-permeable agent significantly increases after oxidation. Cultured HepG2 cells were stimulated using FFA (1 mM) with or without pre-incubation with Ang-(1–7) (10 nmol/L) or A779 (1 μmol/L). After 1 h, 5 μmol/L DCF-DA solution was added for 40 min at 37 °C in the dark. The cells were washed in phosphate-buffered saline (PBS), trypsinized and resuspended in 1 mL of PBS, and the intensity of the fluorescence was immediately read using a FACScan flow cytometer (Biosciences, San Jose, CA, USA),at 500 nm for excitation and at 530 nm for emission.

### Nile Red Assay

Nile red is an excellent stain for the detection of intracellular lipid droplets by a fluorescent microscopy or flow cytometry. The intensity of fluorescence represents the number of lipid droplets in cells. Comparing with oil-red O, Nile-red O staining has higher accuracy, and the operation of Nile-red O staining is simpler. The lipid content in cultured cells was quantified fluorometrically using Nile red. HepG2 cells were seeded at 3 × 10^5^ cells/well into 12-well plates and then treated with 10 nmol/L Ang-(1–7) or 1 μmol/L A779 in the presence of Nile red solution at a final concentration of 200 ng/mL in PBS for 30 min. The intensity of the fluorescence was immediately read using a FACScan flow cytometer (Biosciences, San Jose, CA, USA), at 488 nm for excitation and at 550 nm for emission. Data from 10,000 single cell events were collected. To rule out false-positives, drug treatment in the absence of Nile Red was measured and taken as the background. The relative fluorescence intensities minus the background levels were used for data analysis.

### Determination of Triphosphate (ATP) Content

Cultured cells were lysed in a lysis buffer provided by ATP-Lite Assay Kit (Vigorous Biotechnology Beijing). The medium of cultured cells was also collected for ATP determination. The ATP content was measured (nmol) and normalized by protein concentration (nmol/mg) in the same sample and presented as a percentage of the control.

### Western Blot

30–60 μg of protein were separated by 10% SDS-PAGE, transferred to PVDF membranes (Millipore), blocked with 5% non-fat dry milk, and probed with antibodies at 4 °C overnight. The blots were incubated with HRP-conjugated anti-IgG followed by detection with ECL (Santa Cruz). Antibodies against tumor necrosis factor (TNF)-α, monocyte chemotactic protein-1 (MCP-1), interleukin-8 (IL-8) and Akt were all purchased from Cell Signaling. Acetyl-CoA carboxylase^44^, sterol regulatory element-binding protein (SREBP)-1c, liver X receptor-α (LXRα), fatty acid synthase (FAS), uncoupling protein-2 (UCP-2), superoxide dismutase (SOD) 2, glutathione peroxidase (Gpx) 1, catalase and adiponectin receptor 1 (AdipoR1) were obtained from Santa CruZ.

### Statistical Analysis

All of the data are presented as the mean ± SD. The data were analyzed by Student’s t test or one-way ANOVA (with Bonferroni post-hoc tests to compare replicate means) when appropriate. Statistical comparisons were performed using Prism5 (GraphPad Software, San Diego, CA). P values less than 0.05 were considered to be statistically significant. Representative results from at least three independent experiments are shown unless otherwise stated.

## Additional Information

**How to cite this article**: Cao, X. *et al.* Angiotensin-converting enzyme 2/angiotensin-(1–7)/Mas axis activates Akt signaling to ameliorate hepatic steatosis. *Sci. Rep.* **6**, 21592; doi: 10.1038/srep21592 (2016).

## Supplementary Material

Supplementary Information
