# Supplementary material for: Angiotensin-converting enzyme 2/angiotensin-(1–7)/Mas axis activates Akt signaling to ameliorate hepatic steatosis
Source: Sci Rep. 2016 Feb 17;6:21592. doi: 10.1038/srep21592 (PMC4756304; doi:10.1038/srep21592)
Supplement: Supplementary Information [file srep21592-s1.pdf]

Angiotensin-converting enzyme 2/ angiotensin-(1–7)/Mas axis activates Akt signaling to ameliorate hepatic steatosis

Xi Cao[1,2], Fangyuan Yang[1,2], Tingting Shi[1,2], Mingxia Yuan[1,2], Zhong Xin[1,2], Rongrong Xie[1,2], Sen Li[1,2] , Hongbing Li[1,2] & Jin-Kui Yang[1,2]*

Supplementary Figure. 1

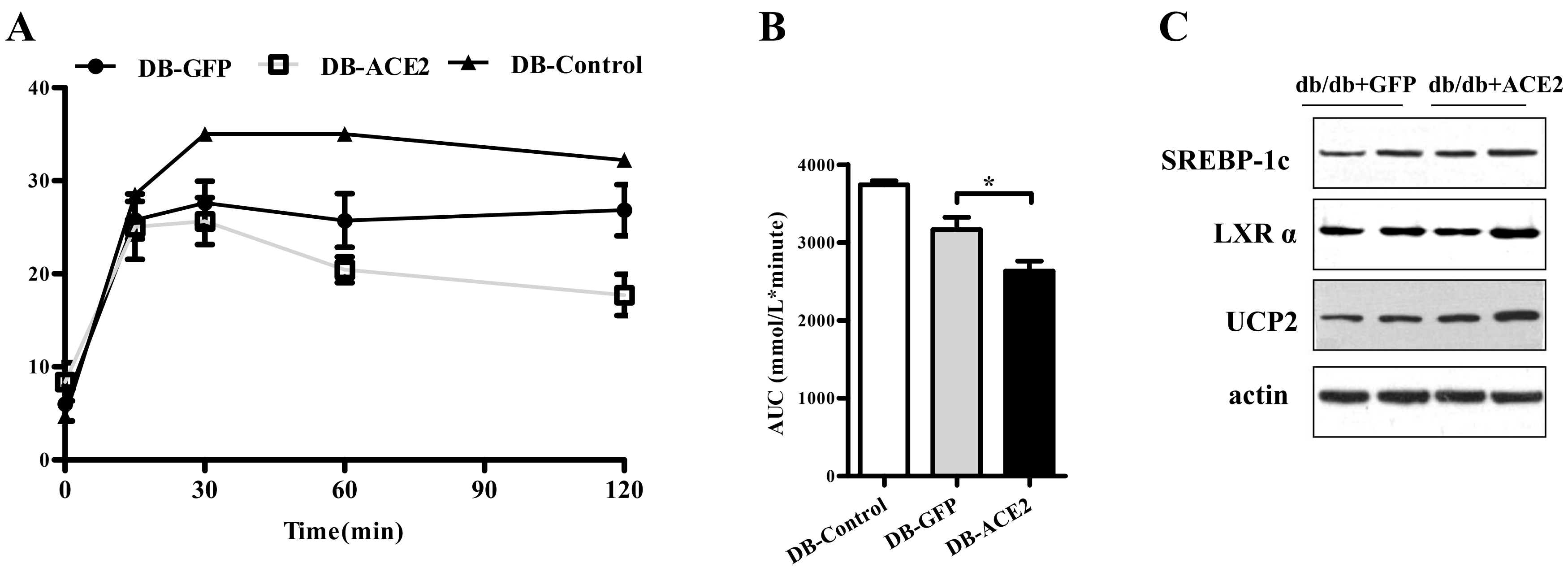

Figure S1. Hepatic overexpression of ACE2 ameliorated hyperglycemia of db/db mice. (A) OGTT of mice at 7th day post Ad-GFP or Ad-ACE2 injection. Area under curves (AUC) for the OGTT data is shown in (B). (C) Relative gene expression levels of lipogenesis genes (SREBP-1c, LXR α and UCP-2). The data are presented as the mean ± SD of n=4 independent experiments in ACE2-overexpressing db/db mice. *P<0.05 versus db/db+GFP by Student's t test.

Supplementary Figure. 2

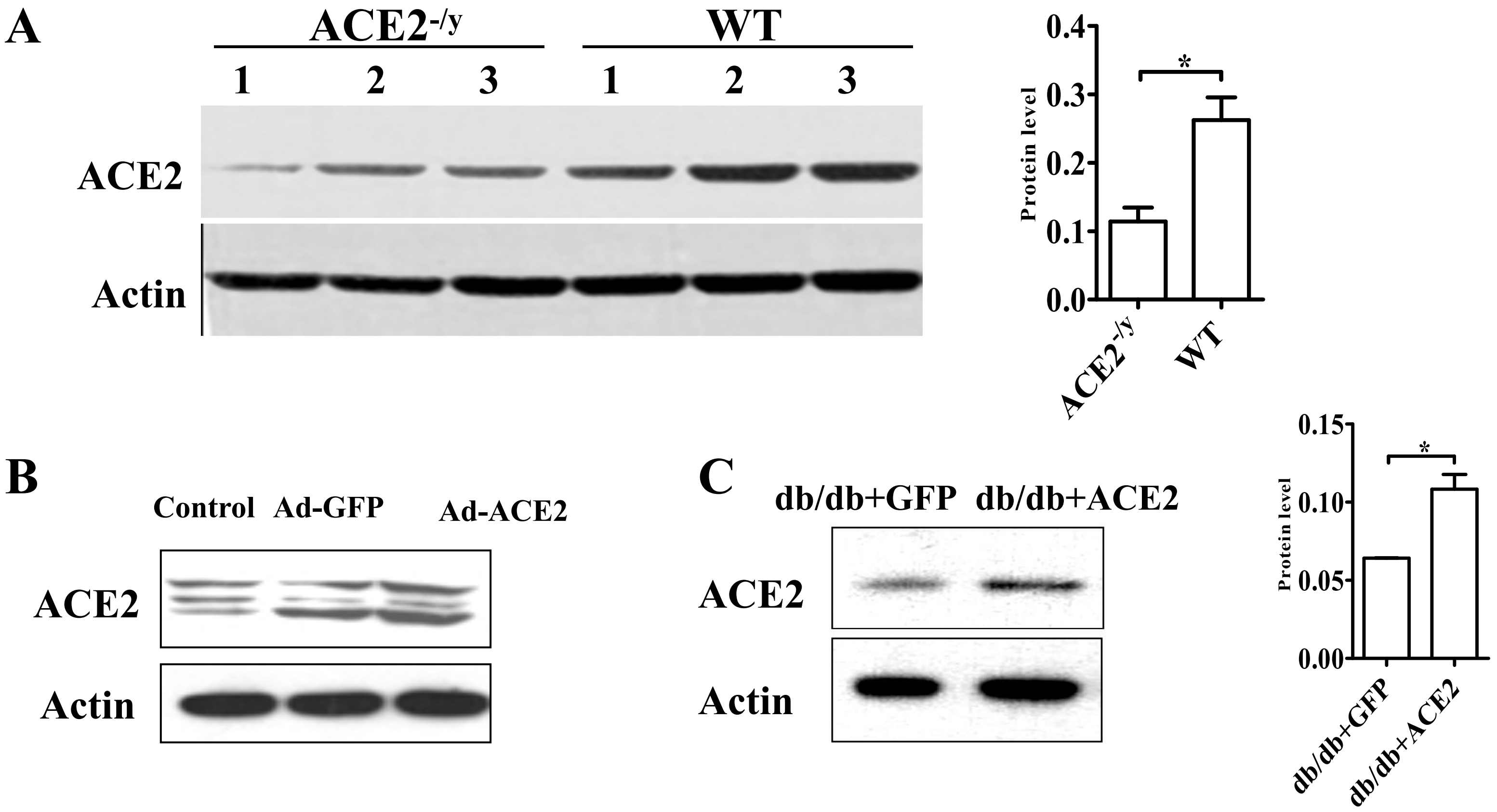

Figure S2. The expression of ACE2 in ACE2-overexpressing HepG2 cells. (A) The protein level of ACE2 in the liver of ACE2 KO mice. (B) Representative gel images of ACE2 in ACE2-overexpressing HepG2 cells. (C) The protein level of ACE2 in the liver of ACE2 injected db/db mice.
